# Medawar's legacy to cellular immunology and clinical transplantation: a commentary on Billingham, Brent and Medawar (1956) ‘Quantitative studies on tissue transplantation immunity. III. Actively acquired tolerance’

**DOI:** 10.1098/rstb.2014.0382

**Published:** 2015-04-19

**Authors:** Elizabeth Simpson

**Affiliations:** Division of Immunology and Inflammation, Department of Medicine, Imperial College, Hammersmith Campus, Du Cane Road, London W12 0NN, UK

**Keywords:** transplantation, tolerance, cellular immunity, histocompatibility

## Abstract

‘Quantitative studies on tissue transplantation immunity. III. Actively acquired tolerance’, published in *Philosophical Transactions B* in 1956 by Peter Medawar and his colleagues, PhD graduate Leslie Brent and postdoctoral fellow Rupert Billingham, is a full description of the concept of acquired transplantation tolerance. Their 1953 *Nature* paper (Billingham RE *et al*. 1953 *Nature* **172**, 603–606. (doi:10.1038/172603a0)) had provided initial evidence with experimental results from a small number of neonatal mice, with mention of similar findings in chicks. The *Philosophical Transactions B* 1956 paper is clothed with an astonishing amount of further experimental detail. It is written in Peter Medawar's landmark style: witty, perceptive and full of images that can be recalled even when details of the supporting information have faded. Those images are provided not just by a series of 20 colour plates showing skin graft recipient mice, rats, rabbits, chickens and duck, bearing fur or plumage of donor origin, but by his choice of metaphor, simile and analogy to express the questions being addressed and the interpretation of their results, along with those of relevant published data and his prescient ideas of what the results might portend. This work influenced both immunology researchers and clinicians and helped to lay the foundations for successful transplantation programmes. It led to the award of a Nobel prize in 1960 to Medawar, and subsequently to several scientists who advanced these areas. This commentary was written to celebrate the 350th anniversary of the journal *Philosophical Transactions of the Royal Society*.

## 1. Summary of the findings

**Figure 1.**
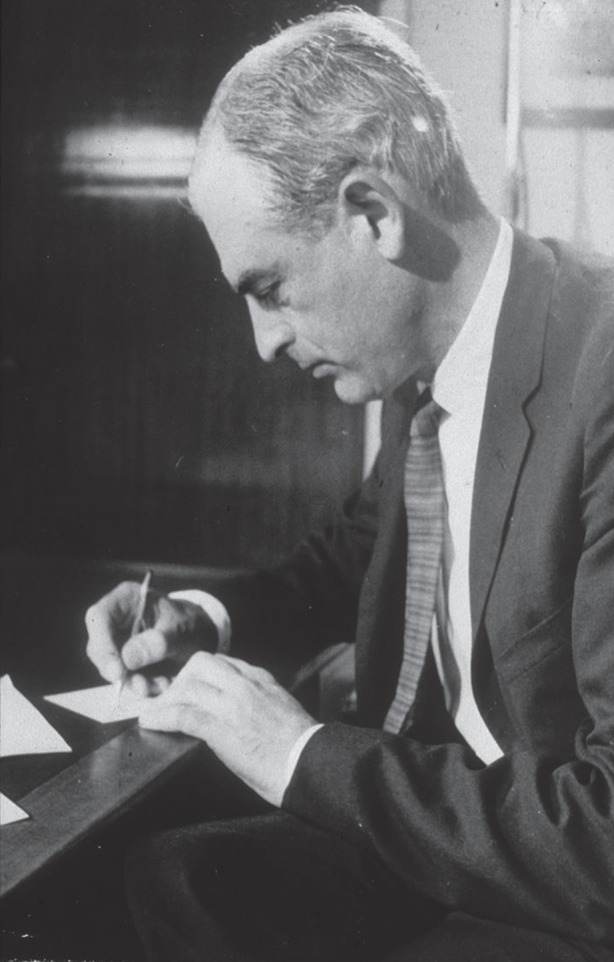
Peter Medawar in 1960, answering congratulatory letters for his Nobel Prize.

**Figure 2.**
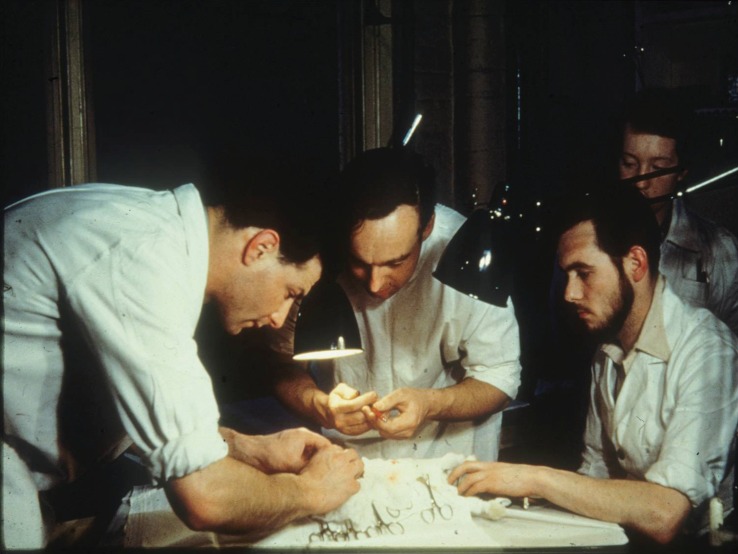
Leslie Brent, Rupert Billingham and technician Trevor Courtenay at University College London in 1955/1956, working out the intravenous route for the inoculation of cells into newborn mice. (Image provided by Leslie Brent).

The story of this work done by Peter Medawar (figure 1) and his colleagues, PhD graduate Leslie Brent and postdoctoral fellow Rupert Billingham (figure 2), was sparked by an unexpected result of skin grafting experiments in twin cattle [1,2]. Medawar's earlier research had focused on the rejection of skin grafts by burns patients [3,4], using outbred rabbits to investigate the process [4–7]. This research identified immune responses characterized by lymphocyte infiltration of genetically dissimilar grafts (but not of autografts, which healed in and remained) as being responsible for rejection in both species, and by implication in others. Subsequent exposure to grafts from the same donor resulted in faster rejection times, a characteristic of immunological memory. Medawar was later asked by the Animal Breeding Research organization to determine whether exchange of skin grafts between cattle twins could distinguish between identical (monozygotic) and fraternal (dizygotic) pairs. To his surprise, dizygotic as well as monozygotic recipients retained their test twin grafts, while still able to reject ‘third party’ grafts from unrelated donors [1,2].

**Figure 3.**
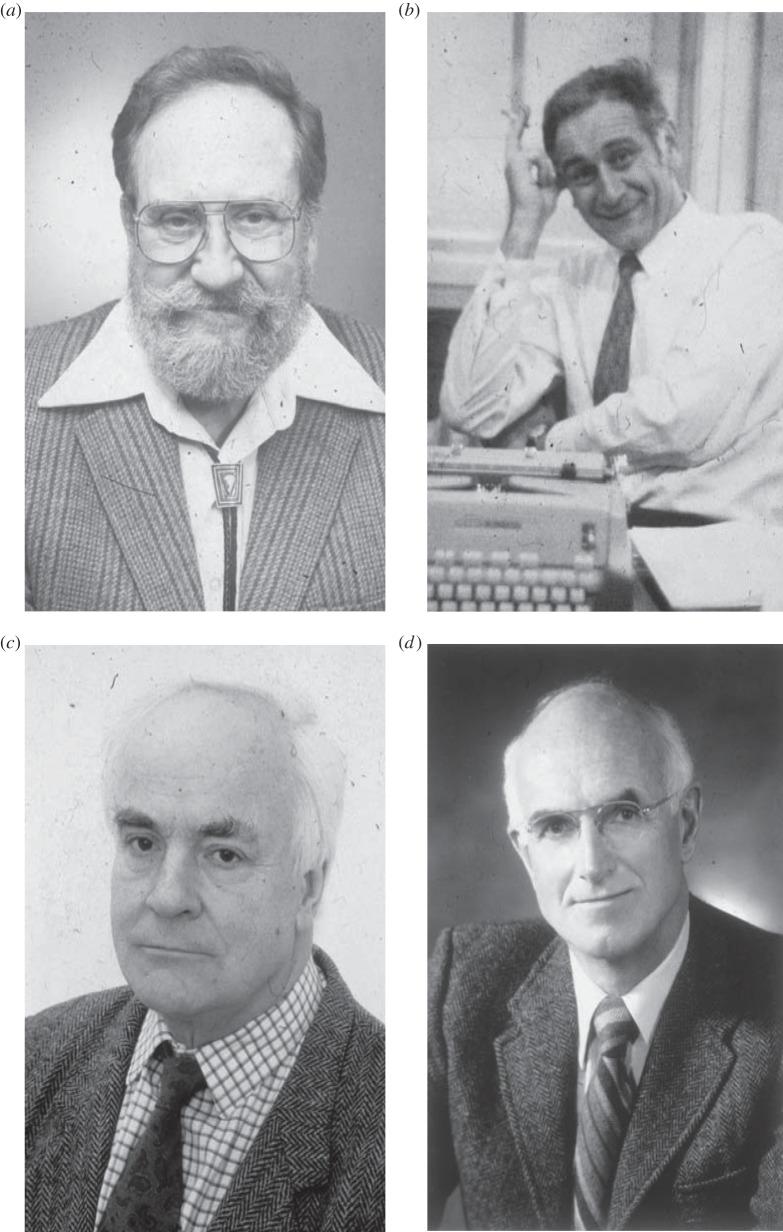
Influences and collaborators. (*a*) Ray Owen discovered that chimaerism in cattle twins protected RBCs of both animals in each of them; (*b*) Milan Haŝek, a Czech immunologist, friend and colleague of Peter Medawar; (*c*) Avrion Mitchison, a former student of Medawar's who pioneered functional cellular and molecular analyses of T helper cells; (*d*) Joseph Murray, who carried out the first human kidney transplants.

Through Burnet's writing on theoretical aspects [8], he became aware of how immune responses might distinguish ‘self’ from ‘non-self’ (i.e. ‘foreign’). Burnet's hypothesis was influenced by a report from Ray Owen (figure 3) that, as adults, twin cattle had circulating red blood cells (RBCs) bearing genetic markers of both twins [9]. Owing to a shared placental circulation they became chimaeras with respect to erythropoietic stem cells (the half-life of RBCs is short, requiring constant replenishment from stem cells). This type of chimaerism is common in cattle, but rare in twins of other placental species, including sheep and humans. RBC chimaerism and weakened immune responses to their parabiotic partners had been reported in birds by Hašek (figure 3) [10,11], who at the time gave the results a politically expedient interpretation supporting Trofim Lysenko's views denying attribution to genetic control, influenced by Lamarck and supported by Stalin.

Reproducing the essential elements of chimaerism in inbred experimental species appeared to Medawar's team to be a perfect way of exploring the effects of early life exposure to tissues from a genetically dissimilar individual with respect to immunological responses to non-self antigens. The effects could then be tested during the recipient's adult life by grafting skin from a donor genetically identical to the original tissue source, and thus address Burnet's hypothesis.

**Figure 4.**
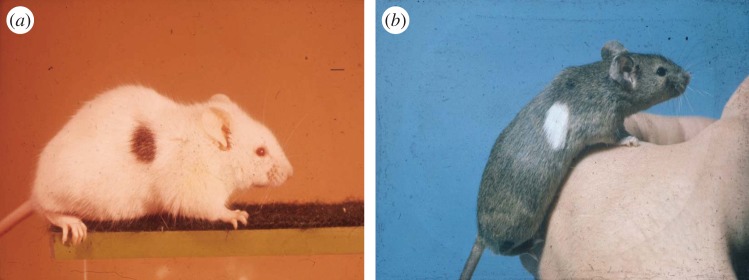
Mice with skin grafts from genetically dissimilar donors (‘homografts’, now termed ‘allografts’) with differently pigmented skin appendages (fur).

The short report of their initial findings was published in *Nature* [12], with a more extensive account in their landmark paper in *Philosophical Transactions B* [13]. The alphabetical order of their names in publications was Medawar's convention and acknowledgement of their team work. They found that following the injection of late-stage mouse embryos [13] or neonates [14] of an inbred strain with cell suspensions from another strain, test skin grafts placed on them as young adults were not rejected: a significant proportion of the recipients had been rendered tolerant indefinitely (‘fully tolerant’), accepting the foreign grafts as ‘self’ (figure 4). This was powerful support for Burnet's ideas, and opened up the possibility that, akin to induction of protective immune responses to pathogens by vaccines, introduction of antigen to an immature immune system was an alternate path through which tolerance could be acquired through an immune response.

Tolerance induced in this way was immunologically specific: test skin grafts from mice of other inbred strains (‘third party grafts’) were rejected, leaving unaffected the grafts from the donor-strain whose tissues had been injected into the embryos. Since various cells, including lymphocytes, could be used to induce tolerance by injection into embryos and neonates, it was also clear that the transplantation antigens on skin were expressed in a wide variety of tissues.

Stability of the tolerant state in these mice was tested by ‘adoptive transfer’ of immuno-competent cells, i.e. injecting fully tolerant recipients either with lymphocytes from naive mice of the same strain as the tolerant animal, or with ‘memory’ lymphocytes from mice immunized with tissue from the strain of the test skin graft donor. These experiments used the method devised by Avrion Mitchison (figure 3), a former PhD student of Medawar [15]. The memory lymphocytes induced a rapid rejection of previously tolerated grafts, while rejection occurred more slowly after adoptive transfer of lymphocytes from naive donors. Susceptibility to rejection was evidence for the tolerated grafts not having lost expression of target transplantation antigens, since they were still vulnerable to attack. This argued against ‘graft adaptation’ as an explanation for tolerance acquired neonatally.

These experiments demonstrated two further characteristics: firstly, fully tolerant recipient mice could be said to show ‘central failure’ of their own response to the tolerated transplantation antigens (according to Burnet's theory, caused by clonal deletion of reactive cells), and secondly, that the mice had not developed the means of preventing potentially graft-rejecting lymphocytes transferred into them from becoming effector cells. A rider to this last conclusion was however considered with respect to partially tolerant mice, i.e. those who had retained their grafts beyond the normal primary rejection time, but showed eventual breakdown and slow rejection of the graft. In the discussion, the possibility was raised that such mice might have developed a state of regulated balance of effector cells. In retrospect, this is prescient, but that is a scenario to be discussed later.

The 1956 paper set the scene for showing that, as with late-stage fetuses made tolerant by *in utero* injections of various tissues from another strain, newborn mice could similarly be rendered tolerant by intravenous injection of lymphocyte suspensions from the other strain [14] (figure 4). Importantly, this tolerance could only be induced during the first few days after birth. This narrow window was followed by a brief ‘null’ period when neither tolerance nor immunity was induced, while if injections were delayed a few more days the effect was to enhance immune responsiveness, resulting in more rapid rejection of test skin grafts.

**Figure 5.**
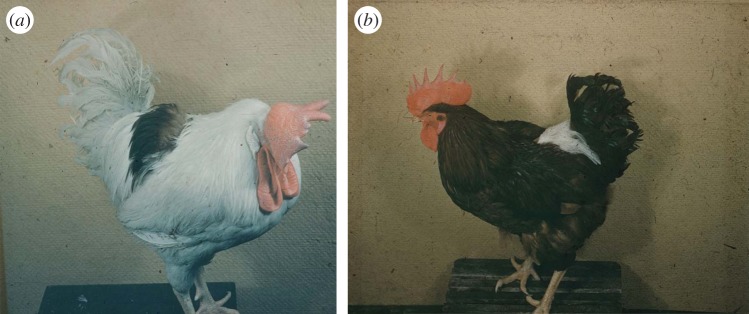
Homografts (allografts) on birds which were synchorially united to their future donors during embryonic life. (*a*) White Leghorn cockerel with skin graft from his Rhode Island Red parabiotic partner; (*b*) Rhode Island Red hen with skin graft from her White Leghorn partner.

The probable role of chimaerism in maintaining tolerance to the test skin grafts was confirmed in experiments with chicks injected *in ovo* with cells from another bird, since erythrocytes in birds are nucleated and retain cell surface expression of antigens detectable by appropriate antisera (figure 5). Induction of transplantation tolerance by injection of embryos with donor cells from another member of the same species was also found in rabbits and bird species such as ducks, but as both were outbred such experiments were more difficult to perform, because the donor of the tolerance-inducing tissue had to be kept alive to provide the test skin graft.

Finally, but only in the discussion of this paper, the essential paradox of pregnancy was formulated, with speculation about control mechanisms that allow a fetus, despite expressing foreign paternal transplantation antigens, to remain in the mother's uterus during development rather than being rejected in the way that a skin graft of paternal tissue would be.

The earlier experiments leading up to their landmark 1956 paper [13], as well as the subsequent clinical and immunological sides of this scientific story have been tracked down, with great skill, by Daniel Davis in his recent book, *The histocompatibility gene* [16].

## 2. Reception and impact

The *Philosophical Transactions B* 1956 paper and its *Nature* 1953 precursor [12,13] were read with great interest by both fellow immunologists and academic clinicians. These included Donall Thomas, an American haematologist caring for patients with bone marrow failure. He obtained evidence in several of his patients receiving bone marrow allografts of transient donor bone marrow chimaerism, although these patients eventually died from their underlying disease [17]. One of his first patients who was given donor bone marrow had been accidentally exposed to radiation, and others were suffering from leukaemia for which irradiation and/or chemotherapy had proved toxic to their haematopoietic stem cells as well as the tumour. Subsequently, ablative and non-myeloablative protocols were developed to establish bone marrow transplant therapy with long-term chimaerism.

The Boston surgeon Joseph Murray (figure 3) and his colleague Paul Russell, who later spent a sabbatical with Medawar in London, were also influenced by Medawar's findings and knew him personally. Joseph Murray wrote to me in 2005 following the publication of my memoir of Peter Medawar, commissioned by the *American Journal of Transplantation* [18]. In it he reminisced about his personal and working relationship with Medawar, and their early days during which ‘Peter frequently visited the Peter Bent Brigham Hospital. I still recall taking him on rounds visiting some of our early experimental renal transplant patients. He commented that it was the first time he had ever seen transplant patients. In retrospect, it must have been quite different from visiting mice in cages. Peter was always generous in his recognition of the contribution of surgeons to Transplantation Biology, a trait not often found in basic scientists’. Murray carried out the first genetically non-identical human kidney transplants (i.e. from a sibling, non-identical dizygotic twin or an unrelated donor) after successfully transplanting a therapeutic kidney from one member of a pair of identical twins (monozygotic) to the other [19]. At that time, it was known that tolerance could be induced by exposure to appropriate doses of foreign protein antigens, but the findings by Medawar and his small team [12,13] were new: immunological tolerance to transplants could also be acquired. This gave hope for using transplantation as a therapy for end-stage organ failure. Immunological barriers were still there, since the neonatal tolerance results reported made clear that even in experimental mice, susceptibility to tolerance induction diminished with age.

The kidney transplant field initially made progress by employing immunosuppressive drugs to overcome rejection responses in adults. The clinical and experimental work in Boston attracted the talents of the next generation of transplant surgeons for training periods and sabbaticals. These included Roy Calne, who in the 1960s contributed to the development of immunosuppressive drugs in Boston, later returning to set up kidney and liver transplantation programmes in Cambridge, UK. Roy recalls a student asking Peter after a lecture (around 1960) whether his experimental findings using mice had any clinical significance, to which he received the reply, ‘none whatsoever’. Although reasonable at that time, I suspect that Medawar's response may have had layers of meaning: ‘no, not this way’, ‘not yet’ or he was testing the student's imagination. Peter himself remained deeply committed to improving the lot of humans, and jokingly referred to himself as ‘fighting against disease and ignorance’, with a huge smile on his face.

One thing that influenced the route Medawar and his colleagues took was that the first immunosuppressive agents used for kidney transplantation were so toxic, understandably, as they were initially developed for cancer chemotherapy; immunosuppression was a side-effect. However, the patient need was desperate, and their use, together with X-irradiation and cortisone, did prolong the survival of kidney transplants, to a greater extent than would have been predicted by the concurrent laboratory-based research on animals, a point not lost on Calne.

Alongside the clinical work, experimental studies aimed at improving transplantation outcomes were pursued in experimental dogs, rats and mice in research laboratories of both basic scientists and academic clinicians, on each side of the Atlantic and in the antipodes. Medawar had previously investigated, with Billingham *et al*. [20], the immunosuppressive effects of cortisone, but clinically cortisone was not without its own undesirable side effects, so new approaches were needed.

Medawar's Nobel prize award in 1960 was in recognition of the significance of his 1953 and 1956 papers [12,13] on induction of transplantation tolerance, experimentally providing supporting evidence for his co-awardee Burnet's important hypothesis on self/non-self discrimination. For Medawar that work marked not a conclusion, but a way to move forward. At the end of his Nobel prize speech [21, p. 5], there is a telling section enumerating important, as yet unanswered questions raised by the discovery of induced transplantation tolerance. These throw a very interesting light on the state of immunological knowledge at that time:

Far too much is still uncertain. We do not yet know whether any one antibody-forming cell is potentially capable of making any antibody within the organism's immunological repertoire or whether the competence of any one such cell is restricted to a sub-class of the reactions that can be engaged in by the organism considered as a whole. We do not yet know whether the act of synthesis undertaken by an antibody-forming cell is strictly and specifically underwritten by the cell's genetic make-up or whether, in J. Lederberg's terminology, the instructions that govern that act of synthesis are imparted by the antigen itself. And if it should be true that the antigen does no more than choose between one set of preexisting instructions and another, we still do not know whether those instructions are already present in the zygote and therefore part of the legacy of its descendants, or whether they must be added to mutation, necessarily during the course of growth. Finally, we do not even know whether the antitheses as I have put them are wisely so put or not. But it is the study of tolerance that has raised these questions … and which, in due course, will make an important contribution to their answers.

Transplantation Immunology was indeed the starting point for the new branch of cellular immunology, which in turn contributed appropriate experimental systems for pursuing the cellular and molecular biological aspects of B- and T-cell responses.

## 3. Subsequent developments

Peter Medawar remained a key figure in the field, attending meetings of the Transplantation Society and being elected president in 1966, as were Rupert Billingham and Leslie Brent later, when they were chairmen in their respective departments, Billingham at the University of Pennsylvania, USA and Brent at St Mary's Medical School, London. In the USA, Billingham made contributions to reproductive immunology, exploring models for the pathology of two-way immune responses between mother and fetus. The work of both Medawar's and Brent's laboratories focused on basic research to develop biological reagents such as anti-lymphocyte serum (ALS) and to devise new models of tolerance induction in adult mice. Brent found evidence for regulatory T cells in mice made tolerant following treatment with ALS [22]. Medawar investigated the preparation and use of ALS with the young clinicians who came to his laboratory to do PhDs ([23,24]). ALS was subsequently incorporated in some protocols to treat transplant patients and those with autoimmune disease such as multiple sclerosis, before the later development of monoclonal antibodies that ALS foreshadowed. Indeed, polyclonal ALS is still commonly used in some clinical programmes.

In the 1980s, several new immunosuppressive drugs were found as the products of various fungal species and developed further for clinical use by pharmaceutical companies. The first of these was cyclosporine (ciclosporin), although it was to some extent both renal- and hepato-toxic. It was followed by tacrolimus and sirolimus, calcineurin and mTor inhibitors that block activation of T cells. Although these were less toxic they, like all ‘blanket’ immunosuppressive agents, put the recipient at risk of developing infections and some cancers. For this reason, the search for the next generation of biological reagents with similar, but more selective effects than ALS, has been pursued by a generation of scientists. This has been based on Milstein & Kohler's [25] groundbreaking work on monoclonal antibodies. The aim has been to introduce appropriate monoclonal antibodies that could be administered for a limited period of time under conditions that facilitate the development of donor-specific tolerance, a highly desirable feature of the neonatal tolerance described in the Medawar team's 1953 and 1956 papers [12,13]. This has been achieved experimentally by Herman Waldmann and his colleagues [26–28], but clinically it has remained a ‘holy grail’. Although donor-specific tolerance has been found in some patients (more frequently with liver transplants, rarely with kidney transplants), who for various reasons have stopped taking immunosuppressive drugs, the critical factors for establishing such ‘gold standard’ tolerance are not yet understood.

Medawar and his team retained contact with the developing immunological and clinical fields, even in those days without easy communication networks. I am aware, through my discussions with Leslie Brent and Avrion Mitchison, as well as Peter Medawar himself, of their contact with a wide range of immunologists, including those working in relative isolation in Prague, particularly Hašek, whose results were rather casually referred to in the landmark paper. Such contacts were taken further when travel to international meetings there became easier. Hašek and Medawar became good friends despite, or perhaps because of, car journeys taken together at perilous speed on the Czech roads of that era, and being subjected to Hašek's ‘bear hug’. In Peter Medawar's Nobel speech [21] he gives weight to Hašek's 1950s chicken parabiosis experiments that, like his own contemporary ones involving *in ovo* injection of foreign cells in chick embryos, resulted in chimaerism and tolerance [10,11]. Medawar also refers to another result his team obtained following intravenous injection of lymphocytes from donors unrelated to the recipients, that of ‘runting’. Runting was reported independently by Morten Simonsen in 1957 [29], who interpreted the associated splenomegaly in recipients as an attack by immuncompetent lymphocytes on the donor of the recipient, i.e. ‘graft-versus-host’ disease.

## 4. Ideas sparked, highways and blind alleys

While publication of the Billingham, Brent and Medawar papers [12,13] caused much excitement among clinicians treating end-stage organ failure, it also had profound effects on the development of immunology. Until that time, the focus of immunologists was on antibodies (serum immunoglobulins): the molecular basis of their exquisite antigen specificity had not yet been discovered, although much was known about immunoglobulin class-associated functions. The notion of a cell-mediated immunity that did not involve lymphocytes working through immunoglobulin was foreign to many immunologists. Initially Medawar considered it possible that the lymphocyte-mediated graft rejection he saw following adoptive transfer might be delivered by antibodies, although in this case the failure to produce the same effect by injecting serum from immunized mice was difficult to understand. Thus for him ‘cell-mediated immunity’ was identified with lymphocytes themselves.

Serendipitous results from Jacques Miller's investigation of lymphomas opened another door for probing the role of different categories of lymphocytes. Miller had designed experiments to test the role of the thymus in virus-induced lymphogenesis, and discovered that neonatal thymectomy in mice induced profound immunosuppression. He probed this both by skin grafting and by immunization with sheep RBCs to test their ability to make antibodies. These mice failed to reject skin grafts from another mouse strain and had a defective antibody response, limited to the IgM class, to sheep red cells [30]. In parallel, the American clinician Robert Good reported cases of human babies with profound immunosuppression following inheritance of recessive genes associated with the developmental absence of a thymus. They were on the same track at the same time, in the early 1960s. While mice, as experimental animals, were more tractable, the existence of similar findings in patients with mutations affecting thymic development indicated an important conserved feature. The role that cells from the thymus (dubbed ‘T cells’) played in graft rejection was also clear in mice carrying the Nude mutation [31]: homozygotes (*nu/nu*) were unable to reject skin grafts from genetically different mice.

The question of whether the antibody producing cells were a separate lymphocyte lineage from those causing graft rejection and other manifestations of cell-mediated immunity was further explored by immunologists including Claman and his colleagues in the USA, [32] and Miller in Australia [33]. It was also pursued by Mitchison [34] and others working in London at the National Institute of Medical Research (NIMR), where Peter Medawar was appointed Director in 1962. This was 2 years after Medawar and the theoretician Burnet were awarded their Nobel prize on tolerance. The scientists working on lymphocyte populations were in contact with each other at meetings and were frequent visitors at NIMR. They gave seminars, vigorously discussed results and exchanged reagents. Their parallel strands of investigation, using a variety of experimental approaches, including *in vivo* adoptive transfer of genetically marked cells of each lymphocyte type, separately or together, arrived at the same conclusion. Antibody producing cells originating in the bone marrow (B cells) were an entirely separable population from the T cells arising in the thymus. Later work on subpopulations of T cells sprang from this background.

T helper cells (Th cells) both help B cells to optimize antibody responses and drive cell-mediated responses such as graft rejection. Th cells are also involved in the activation of another major T lymphocyte type—cytotoxic T cells (Tc cells) that can kill both engrafted foreign tissues as well as the body's own cells when they are infected by virus (see below).

**Figure 6.**
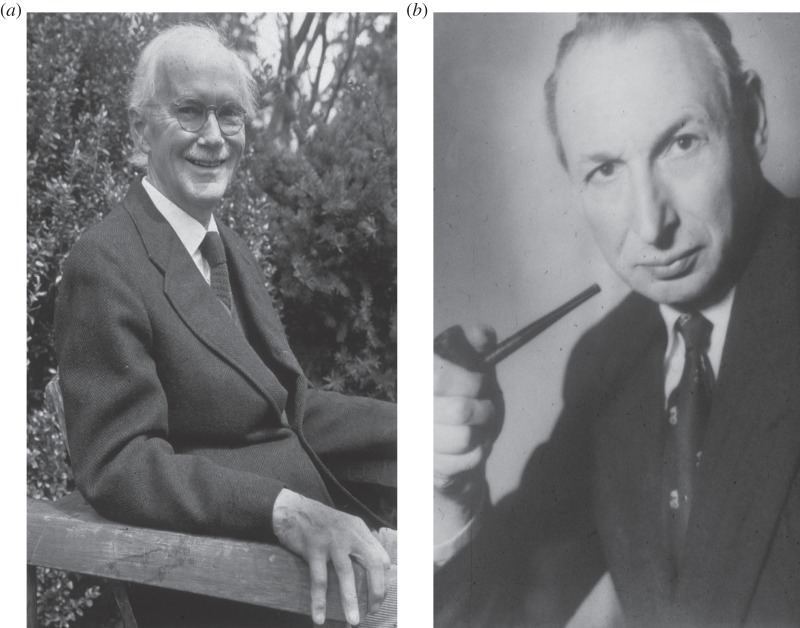
(*a*) George Snell and (*b*) Peter Gorer discovered mouse H2 genes. Gorer died before Snell was awarded a Nobel prize for this work.

**Figure 7.**
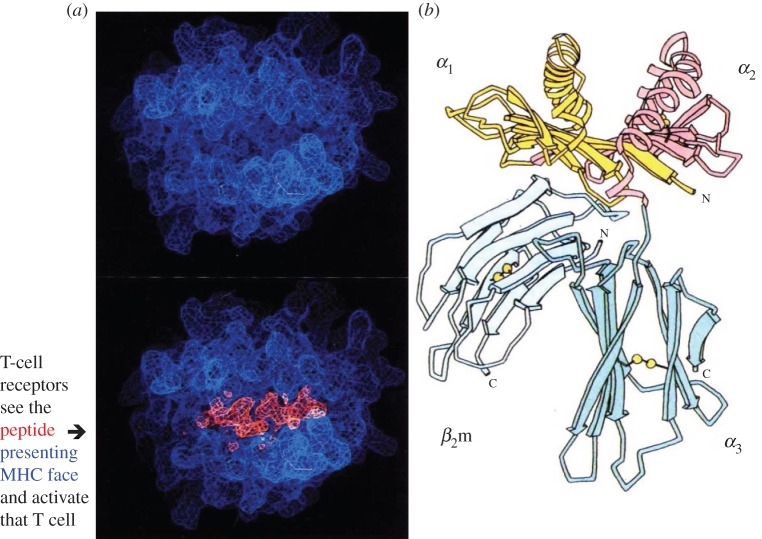
Solution of the crystal structure of an MHC class I molecule showing the membrane-distal domains, *α*_1_ and *α*_2_, folded to create the peptide binding groove. (*a*) Membrane-distal views of the MHC class I molecule HLA-2 without (upper) and with (lower) a peptide fitting into the groove between the *α*_1_ and *α*_2_ helices. (*b*) Same molecule as a ribbon diagram, where *α*_3_ is the membrane proximal-domain of the MHC class I chain while *β*_2_m is a small independently encoded molecule expressed at the cell surface in non-covalent association with the MHC class I heavy chain. Adapted from Bjorkman *et al*. [37], reprinted with permission from Macmillan Publishers Ltd.

A collaborative approach to scientific research was a way of life strongly encouraged by Medawar. He continued working at the bench with his small team to understand further transplantation-related questions. One of those was the molecular identity of the target for rejection. His own earlier work using outbred rabbits, and George Snell and Peter Gorer's (figure 6) research in the late 1940s with inbred mice, had indicated that graft rejection is controlled by a number of independently segregating loci, with one in mice being notably stronger than others. This was designated H2 by Snell [35]. Snell's approach was genetic, generating congenic mouse strains each carrying a single polymorphic histocompatibility locus. Gorer's [36] important contribution was to identify the mouse H2 transplantation antigen with allelic anti-red cell antibodies. These approaches were used by later mouse geneticists to subdivide the ‘strong’ H2 locus into a series of linked genes. Confirmation of this conserved genetic feature was found in humans nearly two decades later by scientists working with serum antibodies from multiparous women and transplant patients. The homologous human locus controlling strong rejection responses, HLA, is also a complex. The term ‘major histocompatibility complex’, MHC, was coined to identify these homologues found in all mammalian and many other species, including birds and reptiles. Medawar [21] had wanted to obtain ‘pure antigen’ for induction of tolerance using biochemical approaches. His and Snell's work on transplantation influenced biochemists like Strominger and Nathenson, who eventually succeeded in purifying HLA and H2 molecules and determining their crystal structure [37,38] (figure 7).

At NIMR Medawar turned his attention to investigating the immunosuppressive qualities of ALS, as mentioned in the previous section. He encouraged and supported scientists at the Institute and recruited an additional number working in disciplines caught up in the spin-out of immunological ideas and questions. It was a heady time. I joined at the end of 1968, and started collaborative experiments contributing to the definition of phenotypically and functionally different subpopulations of T cells [39–41]. I was caught up in the ferment in which microbiologists, cell biologists, physiologists, biochemists, as well as research clinicians in various specialities, moved in and out of each other's laboratories talking about ideas and results. These conversations continued during lunch and coffee breaks, and in the bar at the end of the day. I was encouraged to try new *in vitro* approaches to cell-mediated immunity. I went to the National Institutes of Health (NIH) where I gained experience of growing cytotoxic T cells (Tc) directed against transplantation antigens *in vitro* [42], before the ready availability of growth factors. Analysis of the Tc cell specificity from such cultures led me to results that intersected with those of Zinkernagel and Doherty, working on the other side of the world on T-cell responses to viruses.

Many immunologists in the 1960s and early 1970s, including those working on B cells, regarded questions about how the B-cell repertoire arose as the main frontier for unravelling the specificity of immune responses (see Medawar's Nobel lecture [21]). The antibodies B cells made were accessible from blood samples, and there was a considerable literature about their biochemical properties and functions, which could be investigated following immunization with various antigens. Certain human plasma cell tumours of B lymphocyte origin (myelomas) were found to produce large amounts of monoclonal antibody, a boon for the biochemistry of that time. A strain of inbred mice, BALB/c, had been discovered at NIH by Mike Potter [43] to be susceptible to experimental induction of plasmacytomas (mouse myelomas) that could be serially transplanted into recipients of the same strain, providing a renewable source of monoclonal mouse immunoglobulins. These tumours showed a range of specificities, allowing sequence comparison of their immunoglobulin heavy and light chains. The sequence variants discovered led to speculation about how such differences occurred—was it specified by their respective immunoglobulin genes (germ line) or by somatic mutation? Susumo Tonegawa, then working in Basle, settled this question using the molecular biology methods then being developed. He published his groundbreaking work on somatic gene rearrangement of immunoglobulin gene segments [44]. This process was shown to occur in individual B-cell precursors as they mature from pluripotential haemopoietic stem cells and provides the explanation for the generation of antibody diversity. These findings then formed a model for identifying the similar basis of T-cell receptor specificity.

By then another focus of immunological research had moved to T cells, primed by the cellular definition of transplantation immunity that developed as a direct result of the Billingham, Brent and Medawar paper [13] and by advances in immunogenetics, with molecular biology then waiting in the wings. Work on T-cell subpopulations was carried out with a variety of antigens, including the targets of delayed-type hypersensitivity, transplantation and viruses. Rolf Zinkernagel and Peter Doherty, postdoctoral fellows with medical backgrounds (human and veterinary, respectively) working in Gordon Ada's department in Canberra, were interested in the pathology of neurological disease. Using the mouse virus lymphocytic choriomeningitis virus (LCMV) they discovered, when examining the specificity of LCMV-specific Tc cells, that the target involved both a viral component and one from the infected mouse cells in which it was growing [45].

The mouse component appeared to be associated with transplantation antigens, since only infected cells from mice with the same MHC (H2) alleles were susceptible to lysis. It was their hugely imaginative step to propose that the T-cell receptor recognized two components, separately (two receptors) or together (altered self). They termed this ‘MHC restriction’, implying that all T cells ‘saw’ their cognate antigens in this way. There was a brief resistance to this idea, until an increasing number of immunologists looked closely at the results of their T-cell-mediated responses and discovered independent confirmation. Those working on the category of transplantation antigens designated ‘minor histocompatibility antigens' (minor H antigens) by Snell were among the first to provide this confirmation. My laboratory did so with cytotoxic T cells directed against a single minor H antigen on the Y chromosome, HY [46], and in the same year Mike Bevan, another NIMR alumnus, by then working in the USA, found it was true with multiple unidentified minor H antigens encoded by loci scattered throughout the genome [47]. Later, this was confirmed for single minor H antigens encoded by autosomal genes scattered throughout the genome [48].

Immunologists investigating cytotoxic T-cell responses to additional viruses (e.g. influenza, [49]) in mice and humans, discovered that these Tc cells followed the same MHC pattern, restricted by ubiquitously expressed MHC class I molecules. Similarly, recognition by Th cell is restricted by MHC II molecules, whose expression is limited to a smaller range of cell types. Crucially, these include the cells that process and present intracellular antigens (antigen presenting cells, APC) to Th lymphocytes.

MHC class I and class II molecules are highly polymorphic. They are encoded by different genes in the MHC. Interestingly, the ubiquitous expression of MHC class I genes matches the broad tissue distribution of the antigens found by Billingham, Brent and Medawar in 1956 [13] to be capable of inducing neonatal tolerance. We now know that the MHC class I restricting molecules consist of a single heavy chain, non-covalently linked at the cell surface with a short chain, beta 2 microglobulin (β_2_m), folded together to form a groove to accommodate short peptides (8–11 amino acids) (figure 7). Peptides can be derived from endogenous molecules, the source of minor histocompatibility antigens, or virus growing in the cell. These are the targets recognized by Tc cells.

Crucial information for solving the mystery of how T-cell receptors recognize two components came from the integration of two pieces of information. One was the discovery by Alain Townsend [50] of the short peptide nature of the viral component. The second was the X-ray diffraction pattern of the MHC class I molecule, HLA-A2, crystallized by Pam Bjorkman in 1987 [37]. This structure solved the ‘dual receptor’/‘altered self’ recognition quandary posed by Zinkernagel and Doherty 12 years earlier [45]: both self-MHC and peptide were presented at the cell surface as a complex for the T-cell receptor to recognize. Bjorkman's interpretation of the combined MHC–peptide crystal structure was extended by finding that the polymorphic residues of the class I heavy chain lay in its two membrane distal domains that formed the peptide binding groove [51] (figure 7). The subsequent crystal solution of MHC class II molecules shows their two polymorphic alpha and beta chains are folded together forming a groove capable of accommodating slightly longer peptides. The final pieces in this molecular jigsaw were provided by solving the crystal structures of T-cell receptors (TCRs) from Tc and Th cells restricted by MHC class I or II molecules, respectively, and those of MHC–peptide–TCR complexes [52].

Immune responses as well as tolerance to transplants are MHC restricted. T-cell receptors are exquisitely structured to focus on MHC molecules expressed on the cell surface, class I for Tc cells and class II for Th cells. Both lymphocyte subpopulations need to collaborate for effective rejection responses, like those to molecularly defined MHC class I and II restricted peptide epitopes of the minor transplantation antigen, HY [53–55].

In addition, sub-classes of T cells with regulatory functions (Th1, Th2, Th17, Treg, NKT) have now been identified. Most of these lie within the Th cell, MHC class II-restricted lineage (e.g. [27,55–57]), although NKT cells use other, non-classical MHC molecules [58]. The interaction of all T cell types with cognate antigen associated with its MHC restriction molecule on APC (particularly dendritic cells) is a key event for instigating and maintaining effector and regulatory functions. Regulatory cells have a long and complex history, still being played out. Understanding the mechanisms of immune regulation is important for transplantation tolerance.

## 5. Current relevance and progress

Although this level of detail might be considered beyond the Billingham, Brent and Medawar 1956 paper [13] on induction of transplantation tolerance, each development described above occurred as a direct or indirect result of their earlier work. Does this further defined picture help in solving the transplantation problem? It does, because the underlying immunological principles discovered allow for new approaches. The identity and complexity of interactions between cell types, cell surface, secreted and intracellular molecules has already increased to an extent that could not be envisaged in 1956. That was a time before ideas about and techniques for sequencing DNA, mRNA or RNAi were current, when restriction enzymes had not been discovered, nor DNA methylation and other epigenetic controls of gene expression. Transgenesis by somatic or germline mutation was a distant dream, utopian or dystopian depending on the view. Imaging was limited to classical light microscopy, early electron microscopy and X-ray analysis.

During the ensuing decades, clear evidence has built up that the immune system has evolved under pressure from rapidly mutating pathogens. It is therefore not surprising that it includes an astonishing level of degeneracy (confirmed in many transgenic gene knockout models) to ensure there are many pathways to keep foreign molecules at bay. Rejection of potentially therapeutic transplants is a side effect. Fine control of immune responses employs an array of cell types, cell-bound accessory molecules, lymphokines and chemokines whose function is to orchestrate defence. New molecules and signalling pathways are still being discovered. Clinical advances for improved treatment to transplant patients, as well as those with autoimmune disease and cancer, need to be viewed against this background. They are dependent on both clinical skills and new findings. Some, like the original work on acquired immunity, are triggered by serendipity.

In the clinical field, transplantation has made unprecedented advances in many ways. Hundreds of thousands of kidney transplants have now been performed worldwide, some of those also including the pancreas. A somewhat smaller number of liver transplants, very large numbers of bone marrow transplants, a significant number of heart and/or lung transplants and growing numbers of intestinal transplants have also been carried out. Almost all organ (but not all bone marrow) transplants coming from non-identical donors require the recipient to receive long-term immunosuppression, with its attendant disadvantages. Nevertheless, there have been significant improvements in the choice of agents, based on more detailed understanding of molecular pathways (including antibodies to co-receptors or ligands on T cells or APC, and drugs binding to key molecules in signalling cascades). Antigen-specific transplantation tolerance remains a holy grail, to which a small number of apparently stable transplant recipients taken off immunosuppression may yield clues, although that is controversial.

Immunology has tracked into and sparked development of adjacent fields, beginning with immunogenetics. This in turn needed molecular biology in its many guises, from DNA sequencing to expression studies and transgenesis in increasingly sophisticated forms. Cell biology has developed alongside, providing a powerful way to look at mechanisms, but investigations using more complex models are required to test the *in vivo* relevance of the findings, ‘*in vivo veritas*’, both for transplantation and autoimmunity. Pioneering work, reminiscent of Medawar's on ALS [23], has been carried out by Herman Waldmann and his colleagues on making monoclonal antibodies (MAb) to mouse, dog and human lymphocyte surface molecules. This approach is based on selection of candidate MAb using *in vivo* tests in experimental animals, followed by molecularly humanizing MAb for use in patients [26–28,57]. It illustrates the need to turn to immunology and the appropriate use of experimental animals, especially for any manoeuvre that introduces non-self elements, for example, gene and cell therapy. Animal experiments have become more difficult (and costly), mostly for administrative and legislative reasons, not all of which are rational, but such research is crucial for progress, both for acquisition of basic knowledge and for clinical translation.

There are examples of clinical applications going ahead prematurely, sometimes with disappointing or disastrous results, through inefficacy and/or unexpected side effects. Sometimes a hunch has been right, for example, the initial organ transplants with chemical immunosuppression already discussed, and the highly successful use of anti-tumour necrosis factor (TNF)α monoclonals to treat refractory rheumatoid arthritis (RA) [59]. In both these cases, there was some evidence for the new treatment (induction of transplantation tolerance in one, the presence of TNFα among cytokines in the synovial fluid of RA patients for the other), alongside knowledge of possible pitfalls. Risk/benefit analysis favoured action: patients in the 1950s with end-stage kidney failure had a terminal disease, sufferers from treatment-refractory RA are in pain and become increasingly disabled. While applauding these examples, it is essential that scientists and clinicians continue to design experiments and carry out clinical trials so that evidence-based decisions can be made.

Meta-analysis is a powerful tool for pulling out relevant factors, especially those relating to clinical treatments. Systems biology is a maturing field for identifying the most likely molecules, interactions and pathways from appropriate clinical and laboratory-generated data. Biostatistics underlies both, but while carrying out experiments and seeing patients, the imagination of open minded, creative scientists and clinicians often receives clues that provide vital insight about novel approaches. The work of Billingham, Brent and Medawar, and of their closely associated colleagues, attests to that—they all had a ‘hands on’ approach.

## 6. And the future?

Increased understanding of basic biological processes is crucial for unravelling the complexity of immune responses to transplants, viruses, tumours and autoantigens. Relevant are developments in identifying and sequencing molecules of potential interest and importance, be they DNA, RNA or protein. Advanced technologies are likely to bring new knowledge, providing the complex interactions between molecules can be illuminated by both appropriate biostatistics and a sense of reality-grounded whole-animal physiology. Many scientists and clinicians share an optimistic view, while for others it is tempered with concerns that this approach generates overwhelming amounts of data that cloud scientific imagination, and seldom provide understanding about mechanisms.

Vaccine development has been substantial, with effective vaccines against many acute infectious diseases such as mumps, measles, polio, rubella and whooping-cough in near-universal use. Others remain ‘in development’, either because the target viruses have evolved various immune escape strategies (for example, human immune deficiency virus, the cause of AIDS) or the organism is more complex and the best target molecule(s) and/or the critical protective response may not have been defined (for example, TB and malaria). Flu vaccines necessarily remain in development, as they need to deal with a virus that can change its makeup on a year-to-year basis, both by somatic mutation and by a cut-and-paste mechanism using homologous segments of flu viruses from birds or other species of mammals. The possibility of a pandemic started in this way is probably more likely than some other deadly species-hopping viruses, although currently Ebola is challenging that view. Unravelling the complex nature of protective responses to pathogens with the interplay between innate, humoral and cell-mediated mechanisms requires continued research if there is to be a rational approach to developing new preventative and therapeutic approaches.

Effective vaccines against the HPV viruses that can cause warts and cervical cancer are in prophylactic clinical use in the western world, but they are needed far more in the developing world, where the death rate in women from cervical cancer is disproportionately high. This scenario is also true for some other virus-related diseases: the need for Epstein Barr Virus (EBV) vaccine is greater in some African countries, owing to concurrent parasite diseases, than in the west. Despite the elimination of smallpox by vaccination, there have been problems eliminating polio, owing to pockets of misjudged refusal in certain third world countries. In western countries, universal childhood vaccination for common and potentially devastating virus diseases like measles is being put at risk by lack of objectivity and scaremongering.

In clinical transplantation, there have been reports of some kidney and liver transplant patients who have been taken off or have stopped taking their immunosuppressive drugs, nevertheless retaining the graft, although the majority of patients in this category make rejection responses. For bone marrow transplant recipients with closely matched donors, clonal deletion of donor antigen-specific T cells may occur without clinically unmanageable graft versus-host disease after repopulation of their haemopoietic system by donor stem cells. Such patients may be managed successfully without continued immunosuppression. In this situation, donor cell replacement may have seeded the thymus, where self-reactive T cells are removed continuously.

That scenario is unlikely for other organ transplant recipients unless they have been rendered chimeric following a bone marrow transplant from the same donor. In this situation, the patient has bone marrow and a kidney from one source (the donor) but all the somatic tissues are of recipient genotype. There are some clinical data on such chimaerism being successful in kidney graft recipients [60]. An intermediate state of ‘mixed haematopoietic chimaerism’, in which there is bone marrow of both donor and recipient origin following much lesser degrees of recipient bone marrow ablation, has been described in animal models but has not yet been applied in clinical practice.

Otherwise, individuals who have not undergone deletional tolerance, which is less likely in the absence of bone marrow cells, even in patients on immunosuppression, are likely to retain potentially graft-reacting T cells, rather like the ‘partially tolerant’ mice described by Billingham *et al*. [13]. If so, their antigen-specific tolerance would be unstable. There is controversy about whether or not extensive biomarker screening of blood samples from such patients will reveal a signature of tolerance that could identify those for whom immunosuppressive drugs could be withdrawn and which could indicate targets to which other patients could be manipulated to bring them into a state of tolerance [61]. There may be insufficient data for meaningful analysis of this outbred population on the stability of these patients' grafts in the long-term. There is a much higher percentage of liver transplant patients who appear not to need long-term immunosuppression [62], and they may be a better source for determining possible mechanism(s) of graft tolerance. Perhaps this is the best source for the ‘hope of progress’ that Medawar espoused.

## 7. Further reading

The full story of the experiments leading up to their landmark 1956 paper, and the subsequent clinical and immunological breakthroughs, is discussed by Daniel Davis in his recent book, *The Histocompatibility Gene* [16]. His account is informed not only by published papers and reports, but also from his interviews with family, friends and colleagues. These include the surviving member of the trio, Leslie Brent, whose PhD experiments (thesis now in the archive of the British Transplantation Society), provided much of the data presented in this landmark paper.

Av Mitchison's biography of Peter Medawar [63] is another important source of information.

## Author profile

**Elizabeth Simpson** studied natural sciences and veterinary medicine in Cambridge. Following a short period in clinical practice in Canada, she undertook postgraduate training in pathology, becoming interested in immunological responses to tumours, where good prognoses were associated with lymphocyte infiltration. Medawar's description of skin graft rejection was similar, raising questions about shared mechanisms, i.e. serum factors (antibodies) or lymphocytes (cell mediated). She joined Medawar's team at the National Institute for Medical Research. There, and subsequently as a visiting fellow at NIH, she contributed to the development of cellular immunity. Returning to an MRC post in London, she exploited novel *in vitro* approaches to study immune responses to genetically weak transplantation antigens, discovering that they, like T-cell responses to viruses, were ‘MHC restricted’, recognizing non-self components presented by self-molecules, a principle that applies to all T-cell responses, including those to tumour- and auto-antigens.
